# Supplementary material for: Antioxidant and Anti-Inflammatory Properties of Conceivable Compounds from Glehnia littoralis Leaf Extract on RAW264.7 Cells
Source: Nutrients. 2024 Oct 27;16(21):3656. doi: 10.3390/nu16213656 (PMC11547663; doi:10.3390/nu16213656)
Supplement: Supplementary file 1 [file nutrients-16-03656-s001.zip › nutrients-3250037-supplementary.pdf]

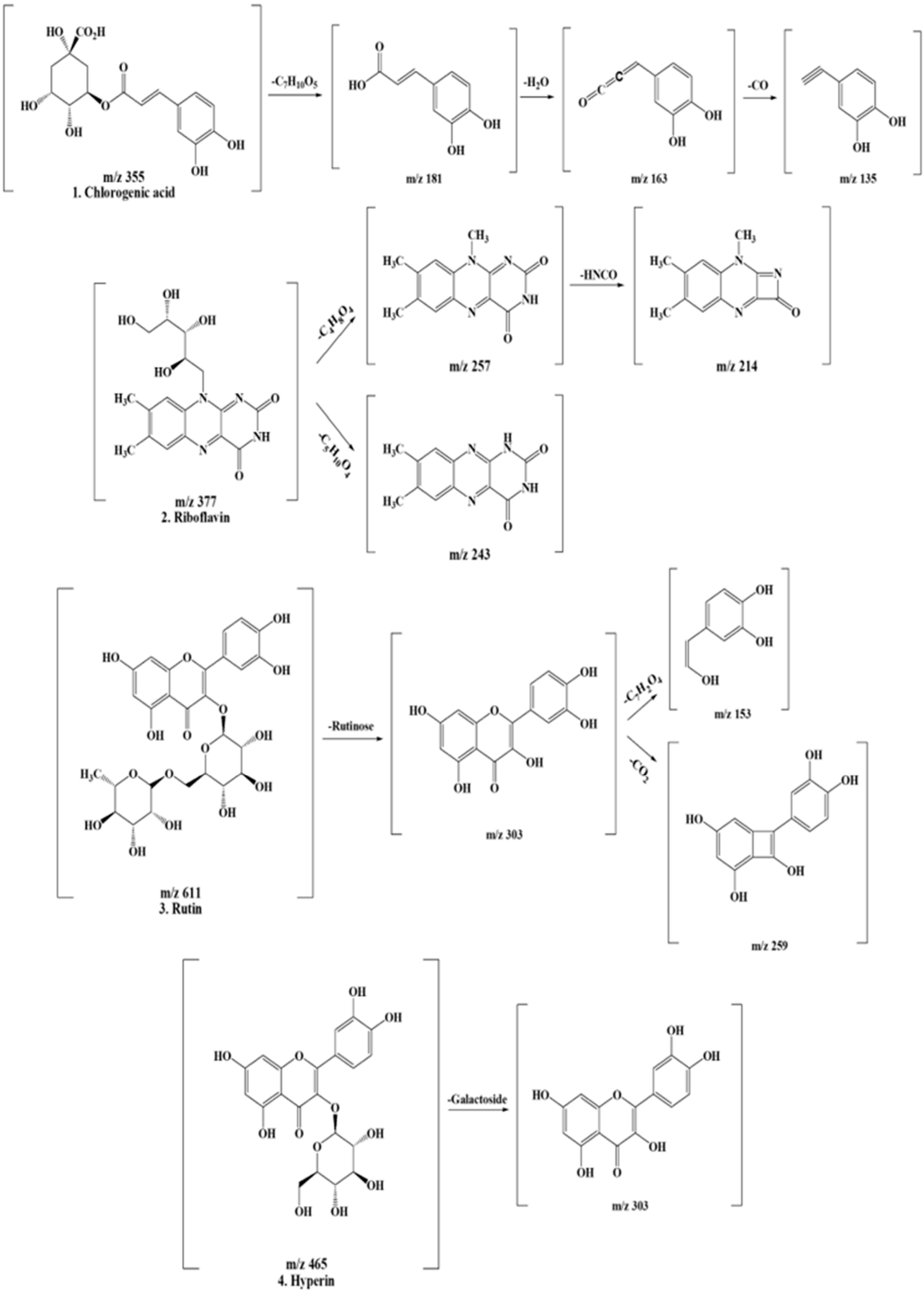

m/z 355
1. Chlorogenic acid
-C7H10O5
m/z 181
-H2O
m/z 163
-CO
m/z 135
m/z 377
2. Riboflavin
m/z 257
-HNCO
m/z 214
m/z 243
m/z 611
3. Rutin
-Rutinose
m/z 303
m/z 153
-CO2
m/z 259
m/z 465
4. Hyperin
-Galactoside
m/z 303

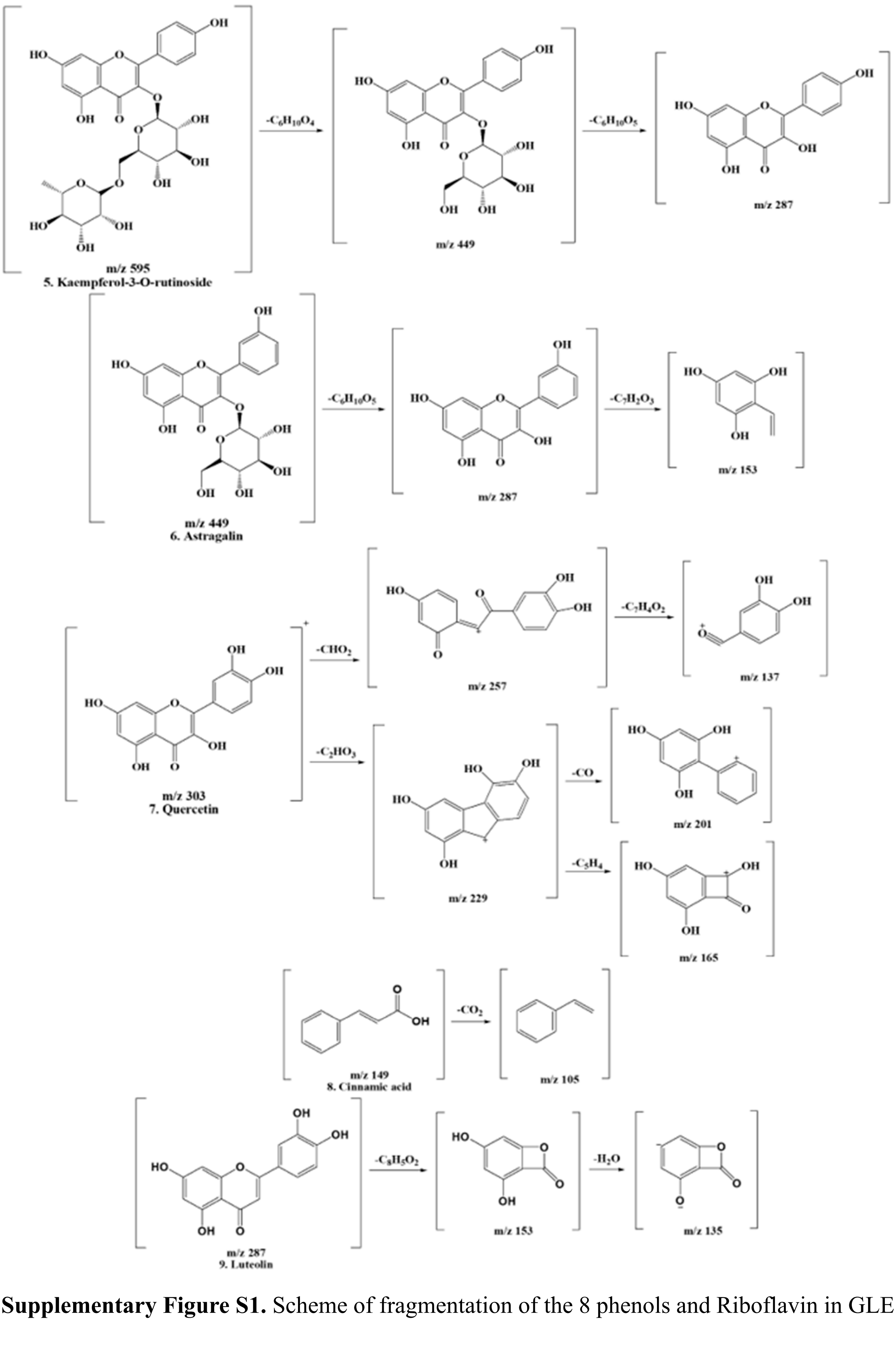

Supplementary Figure S1. Scheme of fragmentation of the 8 phenols and Riboflavin in GLE

Supplementary Table S1. The phenolic compounds and Riboflavin HPLC-MS/MS data from GLE.

Peak No.	Rt (min)	Formula	Compound	UV max	$[M+H]^+$	MS/MS
1	17.44	$C_{16}H_{18}O_9$	Chlorogenic acid	325, 250	355.31	181 ($C_9H_8O_4$) $[M+H-C_7H_{10}O_5]^+$<br>163 ($C_9H_6O_3$) $[M+H-C_7H_{10}O_5-H_2O]^+$<br>135 ($C_8H_6O_2$) $[M+H-C_7H_{12}O_6-CO]^+$
2	21.32	$C_{17}H_{20}N_4O_6$	Riboflavin	440, 365	377.4	257 ($C_{13}H_{12}N_4O_2$) $[M+H-C_4H_8O_4]^+$<br>243 ($C_{12}H_{10}N_4O_2$) $[M+H-C_5H_{10}O_4]^+$<br>214 ($C_{12}H_{11}N_3O$) $[M+H-C_4H_8O_4-HNCO]^+$
3	30.18	$C_{27}H_{30}O_{16}$	Rutin	360, 260	611.52	303 ($C_{15}H_{10}O_7$) $[M+H-C_{12}H_{20}O_9]^+$<br>259 ($C_{14}H_{10}O_5$) $[M+H-C_{12}H_{20}O_9-CO_2]^+$<br>153 ($C_8H_8O_3$) $[M+H-C_{12}H_{20}O_9-C_7H_2O_4]^+$
4	32.23	$C_{21}H_{20}O_{12}$	Hyperin	355, 255	465.4	303 ($C_{15}H_{10}O_7$) $[M+H-C_6H_{10}O_5]^+$
5	34.53	$C_{27}H_{30}O_{15}$	Kaempferol-3-O-rutinoside	345, 265	595.5	449 ($C_{21}H_{20}O_{11}$) $[M+H-C_6H_{10}O_4]^+$<br>287 ($C_{15}H_{10}O_6$) $[M+H-C_6H_{10}O_4-C_6H_{10}O_5]^+$
6	36.70	$C_{21}H_{20}O_{11}$	Astragalin	350, 265	449.4	287 ($C_{15}H_{10}O_6$) $[M+H-C_6H_{10}O_5]^+$<br>153 ($C_8H_8O_3$) $[M+H-C_6H_{10}O_5-C_7H_2O_3]^+$
7	45.94	$C_{15}H_{10}O_7$	Quercetin	375, 255	303.23	257 ($C_{14}H_9O_5$) $[M+H-CHO_2]^+$<br>229 ($C_{13}H_9O_4$) $[M+H-C_2HO_3]^+$<br>201($C_{12}H_9O_3$) $[M+H-C_2HO_3-CO]^+$<br>165 ($C_8H_5O_4$) $[M+H-C_2HO_3-C_5H_4]^+$<br>137 ($C_7H_5O_3$) $[M+H-CHO_2-C_7H_4O_2]^+$
8	47.83	C9H8O2	Cinnamic acid	270, 215	149.16	105 (C_8H_8) $[M+H-CO_2]^+$
9	49.66	$C_{15}H_{10}O_6$	Luteolin	350, 270	287.24	153 ($C_7H_4O_4$) $[M+H-C_8H_6O_2]^+$<br>135 ($C_7H_2O_3$) $[M+H-C_8H_6O_2-H_2O]^+$

RT; retention time

1. Chlorogenic acid

Analysis of a series of chlorogenic acid isomers using differential ion mobility and tandem mass spectrometry

Jamie L. Willems a , Mona M. Khamis b , Waleed Mohammed Saeid b , Randy W. Purves c , George Katselis d , Nicholas H. Low a , Anas El-Aneed b, *

2. riboflavin

Riboflavin degradation products; combined photochemical and mass spectrometry approach. Małgorzata Insinska-Rak ´ a, *, Dorota Prukała a , Anna Golczak a , Emilia Fornal b,1 , Marek Sikorski a, *

3. Rutin

Characterization and Quantification of Phenolic Constituents in Peach Blossom by UPLC-LTQ-Orbitrap-MS and UPLC-DAD. Yu Fu1,2, Ruiqin Sun3 , Jingfan Yang1,2, Lili Wang1,2, Peng Zhao2 , and Suiqing Chen1,2

4. Hypericin

Polyphenols in the Waste Water Produced during the Hydrodistillation of 'Narcea Roses' Cultivated in the Cibea River Valley (Northern Spain). Susana Boso 1, Pilar Gago 1, José-Luis Santiago 1, Inmaculada Álvarez-Acero 2, Miguel-Angel Martinez Bartolomé 2and María-Carmen Martínez 1,*

5. Kaempferol-3-O-rutinoside

Flavonol glycosides and other phenolic compounds in buds and leaves of different varieties of black currant (Ribes nigrum L.) and changes during growing season. Pengzhan Liu a , Heikki Kallio a,b , Baoru Yang a, P. Liu et al. / Food Chemistry 160 (2014) 180–189

6. Astragalin

DPPH Radical Scavenging and Postprandial Hyperglycemia Inhibition Activities and Flavonoid Composition Analysis of Hawk Tea by UPLC-DAD and UPLC-Q/TOF MS. Xuan Xiao 1 ID , Lijia Xu 1 ,

Huagang Hu 2 , Yinjun Yang 1 , Xinyao Zhang 1 , Yong Peng 1,* and Peigen Xiao 1,* Molecules 2017, 22, 1622; doi:10.3390/molecules22101622 .

7. Quercetin

Fourier transform mass spectrometry. Scigelova M, Hornshaw M, Giannakopulos A, Makarov A. Mol Cell Proteomics. 2011 Jul;10(7):M111.009431. doi: 10.1074/mcp.M111.009431.

8. Cinnamic acid

Quantification of flavan-3-ols and phenolic acids in milk-based food products by reversed-phase liquid chromatography-tandem mass spectrometry. Karine Redeuil a, Raymond Bertholet b, Martin Kussmann c, Heike Steiling d, Serge Rezzi a, Kornél Nagy. K. Redeuil et al. / J. Chromatogr. A 1216 (2009) 8362–8370.

9. luteolin

Yuan, L., Zhang, Z., Hou, Z., Yang, B., Li, A., Guo, X., … Li, Y. (2015). Rapid classification and identification of complex chemical compositions in traditional Chinese medicine based on UPLC-Q-TOF/MS coupled with data processing techniques using the KuDieZi injection as an example. Analytical Methods, 7(12), 5210–5217. doi:10.1039/c4ay03103b.
